# Corpus annotation for mining biomedical events from literature

**DOI:** 10.1186/1471-2105-9-10

**Published:** 2008-01-08

**Authors:** Jin-Dong Kim, Tomoko Ohta, Jun'ichi Tsujii

**Affiliations:** 1 Department of Computer Science, School of Information Science and Technology, University of Tokyo, Tokyo, Japan; 2 School of Computer Science, University of Manchester, Manchester, UK; 3 National Centre for Text Mining, University of Manchester, Manchester, UK

## Abstract

**Background:**

Advanced Text Mining (TM) such as semantic enrichment of papers, event or relation extraction, and intelligent Question Answering have increasingly attracted attention in the bio-medical domain. For such attempts to succeed, text annotation from the biological point of view is indispensable. However, due to the complexity of the task, semantic annotation has never been tried on a large scale, apart from relatively simple term annotation.

**Results:**

We have completed a new type of semantic annotation, event annotation, which is an addition to the existing annotations in the GENIA corpus. The corpus has already been annotated with POS (Parts of Speech), syntactic trees, terms, etc. The new annotation was made on half of the GENIA corpus, consisting of 1,000 Medline abstracts. It contains 9,372 sentences in which 36,114 events are identified. The major challenges during event annotation were (1) to design a scheme of annotation which meets specific requirements of text annotation, (2) to achieve biology-oriented annotation which reflect biologists' interpretation of text, and (3) to ensure the homogeneity of annotation quality across annotators. To meet these challenges, we introduced new concepts such as Single-facet Annotation and Semantic Typing, which have collectively contributed to successful completion of a large scale annotation.

**Conclusion:**

The resulting event-annotated corpus is the largest and one of the best in quality among similar annotation efforts. We expect it to become a valuable resource for NLP (Natural Language Processing)-based TM in the bio-medical domain.

## Background

Due to the ever-increasing amount of scientific articles in the bio-medical domain, *Text Mining *(TM) has been recognized as one of the key technologies for future bio-medical research [1-8]. In particular, since the limit of simple TM techniques which treat text as a *bag of words *has become apparent, there has been increased interest in more sophisticated, *Natural Language Processing *(NLP)-based TM. NLP as a field has been engaged in computer processing of structure of a sentence or text. Recently, advanced NLP software which uses grammatical knowledge and/or machine learning techniques has been increasingly applied to TM for the bio-medical domain [9-21].

For NLP techniques to be successfully applied to text in the bio-medical domain, we first have to construct resources specifically designed for NLP in this domain. Since vocabularies are highly dependent on application domains and since text in the bio-medical domain is full of special technical terms, lexicons that associate terms in the domain with their linguistic and semantic properties are indispensable. Recently, several comprehensive lexicons of the domain have been constructed and made publicly available. These lexical resources will bring further progress in NLP-based TM for the domain [22-28].

Less recognized was the necessity of another type of resource for NLP: annotated corpora [29,30]. In the bio-medical domain, not only do special terms appear, but in addition common words are used differently with different meanings. Because of this, we need to re-train or adapt NLP programs for the domain. For example, since the statistical distributions of sequences of part-of-speech (POS) and local syntactic trees are different, POS taggers and syntactic parsers have to be adapted for the domain. For empirical systems, this adaptation requires corpora annotated in terms of POS and syntactic trees. In earlier work, our laboratory constructed such an annotated corpus, the *GENIA corpus*, and made it publicly available to the community [31-33]. Since that time, the GENIA corpus, together with other similar corpora like PennBioIE [34], GENETAG [35], etc., has been used successfully by many groups to develop NLP tools for the domain [36-39].

In this paper, we focus on a new type of annotation, *event annotation*, recently added to the GENIA corpus. Event annotation belongs to what we call *biological annotation*. In contrast with *linguistic annotation *such as POS, or shallow or deep tree annotation, biological annotation is performed by biologists, not by linguists. The goal of biological annotation is to identify what kinds of biological information appear in which part of text, while linguistic annotation focuses on linguistic properties of text in the domain. The term annotation in GENIA is one example of biological annotation. It identifies text spans in which biological entities such as proteins, DNA, RNA, and cellular locations actually appear. As with the POS and tree annotations, the term annotation of the GENIA corpus has been widely used for training NLP tools such as *Named Entity Recognizers *(NERs) [40-44].

Biological annotation is different in nature from linguistic annotation. In linguistic annotation, we can use existing annotation frameworks designed for the general domain, with few changes. In the GENIA corpus, we used the same set of POS tags and phrase tags developed for the Penn Treebank [45]. In contrast, the biological annotation is domain-dependent by definition. For the term annotation, we had to develop our own ontology of term classes (the *GENIA term ontology*) for the domain [46].

Though both the term and event annotations belong to the class of biological annotations, we found event annotation to be much more complicated and challenging than term annotation. Most terms denote ontologically simple entities, e.g. un-analyzable basic units. Even though some entities have internal structures which need a hierarchical structure based on meronymy, their relations are grounded in the concrete physical world. Furthermore, in general, terms appear as continuous spans in text, e.g. according to [32], 98% of terms appear in continuous spans.

An event, on the other hand, is not an un-analyzable unit. It has its own internal structure and it involves biological entities as its participants. The relationships between an event and its participants, those among events themselves such as macro- and micro-events, and the causes of events as well as their consequences, are highly dependent on the conceptualization of events by biologists. The relationship between a macro-event and its micro-events, for example, can be seen as a Part-Whole relation, analogous to a protein and its domains. However, this relationship is far more subtle than those found among physical entities. Furthermore, since each participant in an event is mapped to a span of text, the description of an event as a whole is usually spread over several discontinuous spans in text. Compared with entities denoted by terms, events and their identification in text require much more careful examination in terms of their internal structures and their organization into units. In particular, conception of events and their relationships such as causality reflects an intuitive way of seeing the world. Linguistic expressions of events are strongly affected by this conception. Because our intuitive way of seeing the world is somewhat different from the scientific way of understanding the world, existing biological ontologies alone cannot solve all the ontological issues involved in annotating events in text. We defined the GENIA event ontology, which meets the requirements of text annotation, by referring to existing ontologies, mainly *Gene Ontology *(GO) [47].

The approach to domain-specific event annotation that we adopt in the GENIA project is related to well-known general-domain annotation efforts like Propbank [48,49] and FrameNet [50,51]. All of these projects aim to identify events and their semantic participants in text, however a key difference among them is that they make different assumptions about the relationship between syntactic and semantic annotation.

In PropBank, annotation is performed on the syntactic structures of the Penn Treebank. Annotators find and classify the noun phrases (NPs) that are semantically related to a given verb, and the vocabulary of classes that can be assigned is verb-specific. Some semantic annotation work in biology follows this annotation style [52-54], which demonstrates a progressive analysis of linguistic structures: from constituent structure to predicate-argument structure.

In contrast, FrameNet does not explicitly use constituent structure as the basis for semantic annotation. Instead, the semantic annotation abstracts away from syntactic differences as well as lexical differences. Sentences are labeled using a vocabulary of semantic *frames*, and a group of words share the same frame when they denote the same class of events (e.g. "retail", "sell", and "vend" share the frame of *commerce_sell*).

Since semantic annotation in the FrameNet style abstracts away from syntactic differences, it is closer to the representation we would like to use for text mining. However the frame classification is still based on general-domain frame semantics. Both FrameNet and Propbank annotation styles require annotators who are familiar with their respective linguistic formalisms.

For biological annotation in GENIA, our goal is to use annotators who are biologists, in order to get qualified interpretations from a biological perspective. These annotators are not systematically aware of linguistic phenomena. As a result, our event classification is information-centered, and can be directly mapped to domain knowledge without reference to syntactic or frame-semantic theory.

The disadvantage of this approach is higher inter-annotator discrepancy [55]. While event annotation is performed based on the assertions made in the text, to map the individual assertions with corresponding event classes, annotators depend on inference. Since inference is affected by the annotator's background knowledge, without appropriate control, annotation of the same text may differ from one biologist to another.

In order to minimize discrepancies and maintain the quality of annotation, we have introduced several measures. *Text-bound Annotation *requires annotators to indicate clues in the text for every annotation they make. We have also developed a tool, *XConc*, which provides multi-layered annotation, semantic type checking, and detection of anomalies in the resulting annotations. The annotation guidelines which have been developed during the event annotation also played a key role. The guidelines are written in plain language, and they include many examples of what constitutes appropriate textual evidence for an annotation in GENIA. This helps to define the scope of allowable inferences without using technical definitions that are unfamiliar to our annotators.

After a period of trial and error which lead to initial annotation guidelines, we have completed the annotation of 1,000 abstracts, half of the whole GENIA corpus, with high quality. The annotated corpus consists of 9,372 sentences and 36,114 annotated events, which is by far the largest among similar attempts [53,56]. We will make the annotation results available to the community, and it is reasonable to expect that the corpus will contribute to further progress in NLP-based TM techniques, including event or relation extraction [57-59], intelligent information retrieval [13,60], semantic enrichment of text [61], and integration of text information with pathway databases [11,58].

## Results and Discussion

### Overview of the GENIA corpus

The event annotation presented here builds on our earlier work in extracting the GENIA corpus and annotating it with linguistic features and biological terms. The documents in the corpus come from the Medline database, which covers a broad range of domains in bio-medicine. However, since we are interested in providing semantically rich annotation for text mining in molecular biology, we have focused on a much smaller, semantically homogeneous subject domain: biological reactions concerning transcription factors in human blood cells. We used a search query, *"Humans" [MeSH] AND "Blood Cells" [MeSH] AND "Transcription Factors" [MeSH] *to retrieve a set of articles, and then chose 2,000 of these articles for our annotation.

**Figure 1:**
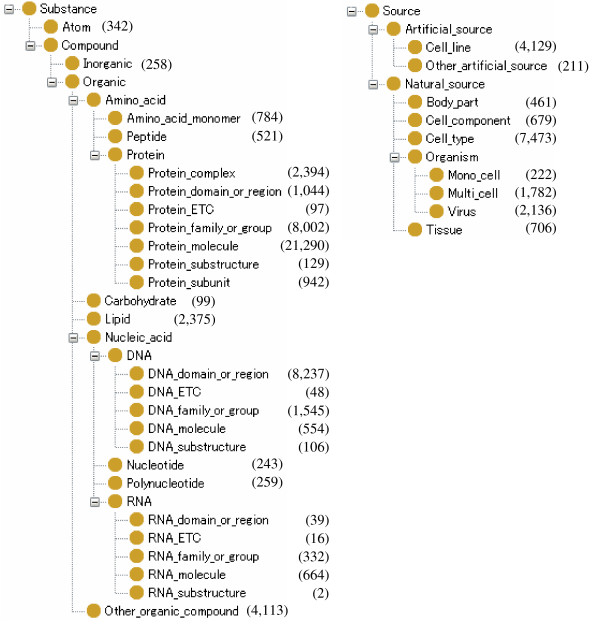
**GENIA term ontology**. The hierarchy of the GENIA term ontology. Terminal classes are used for GENIA term annotation. The figures in parenthesis indicate number of annotation instances made to the GENIA corpus.

The biological annotations in the GENIA corpus include term annotation, which was completed in earlier work, and the event annotation described in this paper. The term annotations include 93,293 bio-medical terms that have been annotated using the 35 terminal classes of the GENIA term ontology (see Figure 1). The event annotation was performed on top of the term annotation, relating the terms.

While terms in text are related with each other in various ways, we have focused on dynamic relations. By "dynamic", we mean that at least one of the biological entities in the relationship is affected, with respect to its properties or its location, in the reported context. Extracting such information from text would be useful in building models of biological systems, e.g. pathways. In order to focus on dynamic relations, some relationships are excluded from our annotation, even though they are biologically important. Static relationships such as Part-of, IS-A, and Similarity relationships between terms are all excluded. (This does not necessarily mean that expressions in text which usually describe static relations were ignored. See Section **Single-facet Annotation **for detail.) Examples of these are given below:

• *The structural similarity of SNI1 to Armadillo repeat protein *... [Similarity]

• *Connexin has four transmembrane domains*. [Part-of]

• *NF kappa B, a transcription factor, is *... [IS-A]

Relationships outside the domain of molecular biology, such as clinical ones involving diseases and symptoms, are also excluded from the current annotation.

### An example of event annotation

**Figure 2:**
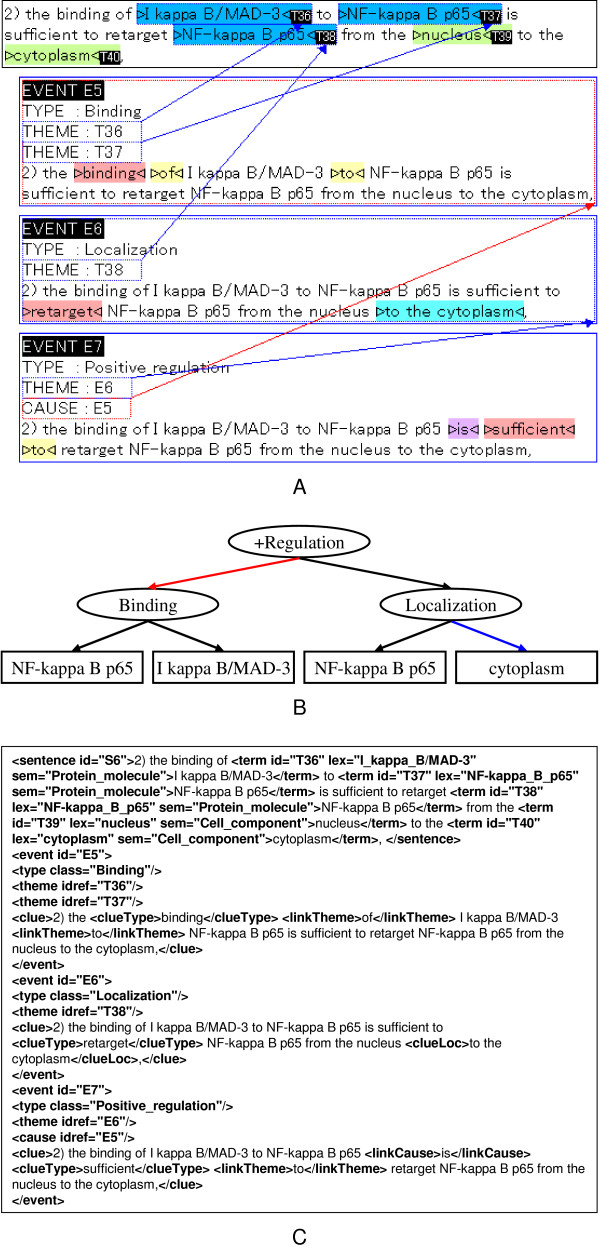
**Example of event annotation**. GENIA event annotation is made sentence by sentence. Although the actual corpus file with annotation is encoded in XML (C), the annotators work on a CSS-styled view (A) which is much more user-friendly. Sometimes, a graphical representation (B) is used to depict annotated events and their relations in an abstract and concise way. Note that the black, red and blue arcs link an event with its themes, causes and location respectively.

Figure 2A shows a screen snapshot of our annotation tool, XConc. There are four regions within the figure, each outlined by a box. The top box contains a sentence under annotation. That is,

*The binding of I kappa B/MAD-3 to NF-kappa B p65 is sufficient to retarget NF-kappa B p65 from the nucleus to the cytoplasm*.

Each of the remaining three boxes displays an event annotation which has been added to this sentence. The original sentence without term annotation is shown inside each of those boxes, to allow annotators to mark-up text spans that belong to the corresponding annotation.

Biological entities, which had been annotated earlier during term annotation, are shown in colors on the screen. Blue and green indicate protein molecules and cell components, respectively. Each term is assigned a term Id (T36~T40 in the example of Figure 2A). These terms are expressed as *n*-tuples of attribute-value pairs as follows:

• (Id: T36, Class: Protein_molecule, Name: *I kappa B/MAD-3*)

• (Id: T37, Class: Protein_molecule, Name: *NF-kappa B p65*)

• (Id: T38, Class: Protein_molecule, Name: *NF-kappa B p65*)

• (Id: T39, Class: Cell_component, Name: *nucleus*)

• (Id: T40, Class: Cell_component, Name: *cytoplasm*)

As mentioned, the three boxes under the input sentence in Figure 2A show three event annotations. The first event E5 represents binding of the two entities, T36 (*I kappa B/MAD-3*) and T37 (*NF-kappa B p65*). The word "binding" is shown in red. This indicates the clue which the annotator used as textual evidence for the existence of a binding event. One of our annotation principles requires each event to be supported by such a clue word. This principle is described in the **Text-bound Annotation **Section. Clue words are described in detail in the section **Linguistic clues and event classes**. Additional supporting words are shown in yellow ("of" and "to").

Each event is also assigned a unique Id. The description of the binding event is:

• (Id: E5, Class: Binding, ClueType: *binding*, Theme: T36, Theme: T37)

The two arguments are specified by their Ids so that they are unique and bound globally over the corpus. The *Theme *in an event is an attribute or slot to be filled by an entity or entities whose properties are affected by the event. The second event E6 represents localization of the protein T38. The textual clues, "retarget" and "to the cytoplasm", are marked up as key expressions denoting the event type and the location relevant to the event, respectively. E6 is represented as:

• (Id: E6, Class: Localization, ClueType: *retarget*, Theme: T38, ClueGoal: T40)

T38 is taken as a Theme since its location is affected by the event. The two entities T37 and T38, though they have the identical textual expressions *NF-kappa B p65*, are distinguished by their Ids. They appear in two different spans in text and thus in different biological contexts. They are identified as the Themes of the two events E5 and E6, respectively. This distinction is important for identification of biological entities in their proper context(See Section **Event annotation and pathways**).

The last event E7 is the causality relation between E5 and E6. That is, the binding event (E5) of the two proteins "causes" the localization event (E6) of one of the two proteins. This causality relation is represented as an event of type *Positive_regulation*.

(Id: E7, Class: Positive_regulation,

   ClueType: *is sufficient to*,

   Theme: E6 (Localization, Theme: T38),

   Cause: E5 (Binding, Theme: T36, Theme: T37))

In the current GENIA event ontology, *Regulation *has a broader definition than regulatory events in a strict biological sense, e.g., *catalysis*, *inhibition*, *up-/down-regulation*, etc. It is used to encode general causality among events. We will discuss the issues related with regulatory events in Section **General causality**. Note that, although the expression *is sufficient to *is hardly a linguistic expression for causality, the annotator recognized it as such in this sentence.

To assist the reader in understanding these relationships, we present Figure 2B, a graphical depiction of the example from Figure 2A. In this representation, entities from the GENIA term ontology are shown in rectangular boxes, while entities from the GENIA event ontology are shown in circles. Black, red and blue arrows indicate a link between an event and its themes, causes and location, respectively.

Figure 2C shows the XML representation of the three event annotations. This format will be used for public distribution of the event-annotated corpus.

### Event annotation and biological ontologies

Although text in natural language (like English) is easy for human readers to understand, the "same" biological events are expressed in diverse surface textual forms. A representation scheme of events such as those in the previous section is important for reducing such surface diversity. It represents the "same" events in the same formats.

In order to establish such a scheme, we have to answer certain ontological questions, such as how to identify the "same" events or the same types of events (event classes), and what structures are needed to represent them. We partly avoided these questions by adopting the Gene Ontology (GO) [47] as our core ontology. We started with GO to define the initial set of event classes and revised them subsequently. The definitions in GO have frequently been referred to by our annotators to judge whether events in text belong to certain event classes or not.

While our information-centered approach to event annotation frees the annotators from linguistics-based criteria for annotation, annotation should not be totally free from text being annotated. Annotation by biologists should be curbed by information actually encoded in text. In other words, annotation should be performed based on information explicitly present in the source text and should not be detached from it too far. This requirement that annotation should reflect the organization of information in text imposes constraints on our representation scheme, distinguishing it from other, more biology-oriented, schema. In the following three sections, we will describe the ontology we used for text annotation, discussing how it differs from other bio-ontologies and the reasons why.

### GENIA ontology and GO

The GENIA event annotation relies on two ontologies: the event ontology and the term ontology. The GENIA event ontology defines and classifies events (or occurrents in the terminology of philosophical ontology [62]) which are of interest in the GENIA domain. In contrast, the GENIA term ontology defines things (or continuants [62]) which cause or run through the events. Roughly speaking, the event ontology provides vocabulary for predicates (e.g. "binding", "phosphorylation", etc.), while the term ontology is for arguments (e.g. proteins) which are used in event descriptions. The term ontology is given in Figure 1. For the details of the term ontology, please refer to [32]. In this section we focus on the details of the GENIA event ontology.

**Figure 3:**
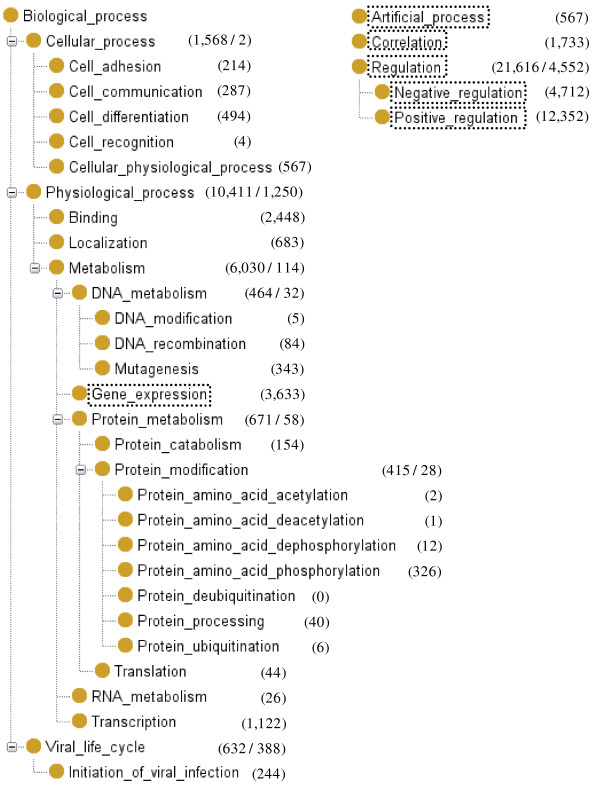
**GENIA event ontology**. The hierarchy of the GENIA event ontology. For event annotation, not only terminal classes but also classes at higher level are allowed to be used. The figures in parenthesis indicate number of annotation instances made to the GENIA corpus.

Figure 3 shows the hierarchy of the event classes in the GENIA event ontology. The numbers attached to the nodes are the number of instances of the events in the current annotation of 1,000 abstracts. With the exception of the six classes shown in dotted boxes (*Gene_expression*, *Artificial_process*, *Correlation*, *Regulation*, *Positive_regulation*, *Negative_regulation*), all event classes are taken from GO. We inherit the names and definitions of the event classes from GO, performing minimal conversion for fitting them into the Web Ontology Language (OWL) naming conventions. While the class of Regulation in GO with its two sub-classes, Negative regulation and Positive regulation, remain in our ontology, the definitions of these classes are different from those of GO (See Section **General causality**). Since the domain of interest in GENIA is much narrower than that in GO, we only use a subset of the GO classes. For example, under the top level class *Biological_process*, we retain only three classes, *Cellular_process*, *Physiological_process *and *Viral_life_cycle*. These three classes reflect the three major topics in the GENIA domain. In particular, *Physiological_process *with its subclasses *Metabolism *and *Localization *is the main focus of the domain. Accordingly, the GENIA event ontology includes the finer grained GO sub-types of these event classes.

In addition, the GENIA event ontology has the following three event classes which GO does not have: *Gene Expression*, *Artificial Process*, and *Correlation*.

#### Gene_expression

**Figure 4:**
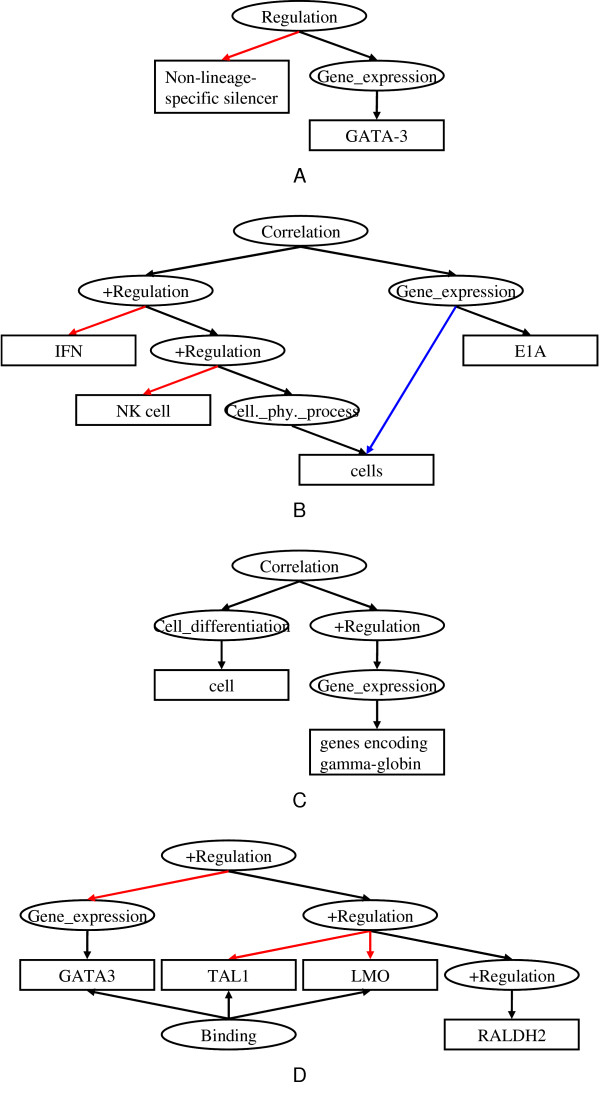
**Graphical representation of events in some example sentences**. Examples in text with corresponding event annotation in graphical representation. (A) *T-cell expression of the human GATA-3 gene is regulated by a non-lineage-specific silencer*. (B) *The extent of IFN-induced NK cell killing of E1A-expressing cells was proportional to the level of E1A expression *... (C) *Cell hemoglobinization was accompanied by the increased expression of genes encoding gamma-globin *... (D) *In addition, forced expression of GATA3 potentiated the induction of RALDH2 by TAL1 and LMO, and these three factors formed a complex in vivo*.

Gene expression is missing from the Gene Ontology, so for the GENIA term Gene_expression, we use the definition given in MeSH, e.g. the phenotypic manifestation of a gene or genes by the processes of genetic transcription and translation. Gene expression is not in GO because it is not a single event, but a macro process. An event in this class consists of micro events or processes such as transcription, translation, and post-translational processes. All of these micro events are in GO. While the decision to exclude a composite process like Gene expression may be justifiable in GO, we need this class for text annotation. The versatility of natural language freely allows authors to express information with variable granularity, and authors often use expressions with coarse granularity to denote complex objects or events. Such expressions are pervasive in text: in the GENIA event-annotated corpus, 3,535 events have been annotated as *Gene_expression*. Some example sentences involving Gene_expression are given below:

• *T-cell ***expression ***of the human GATA-3 gene is regulated by a non-lineage-specific silencer* (Figure 4A).

• *Most retinoblastoma specimens revealed a high COX-2 ***expression**.

• *IL-10 preferentially increased ***expression ***of IFNgamma-inducible genes*.

• *However, B cells can also ***synthesize ***IL-2*.

• *The ability of CMV IE gene products to enhance IL-6 ***production ***may play *...

#### Artificial_process

*Artificial_process *describes experimental processes which are performed by human researchers. Examples include transfection and treatment. Although the use of this event class was not encouraged, the annotators identified 597 events in 1,000 abstracts. Example sentences involving *Artificial_process *are given below:

• ...* to induce NF-kappa B/Rel nuclear activity in cells ***incubated ***in the presence of 3,4-dichloroisocoumarin*, ...

• *Endogenous or exogenously ***administered ***RA may have a significant role in HIV regulation*.

• *Over-expression of STAT2 by ***transfection ***of the cDNA prevented apoptosis of the T cell clones*.

#### Correlation

*Correlation *represents an underspecified relation between events. It is a characteristic feature of natural language that authors can leave irrelevant or unknown details unspecified. Consider the following sentence:

*The extent of IFN-induced NK cell killing of E1A-expressing cells ***was proportional to ***the level of E1A expression *... (Figure 4B)

The text in this example indicates that there is a certain relationship between the two events "*IFN-induced NK cell killing of E1A-expressing cells*" and "*E1A expression*", but the author avoids specifying which event is the cause and which one is the consequence.

Such under-specification is frequently observed in text, and there are many linguistic expressions used to leave the relationships underspecified. While the exact relationship is left unsaid in such expressions, the existence of a relationship between two events is still crucial information for biologists. In these cases we encouraged annotators to use the event type Correlation. 1,722 Correlation events are recognized in the current annotation. Some examples are given below:

• *Cell hemoglobinization was ***accompanied ***by the increased expression of genes encoding gamma-globin*. (Figure 4C)

• *Decreased adhesion molecule expression was ***associated ***with a reduction of monocytic cell adhesion*.

• ...*may have a role in the increase in globin gene transcription that ***characterizes ***erythroid maturation*.

• *This increase in p50 homodimers ***coincides ***with an increase in p105 mRNA*.

### Event annotation and pathways

While developing the annotation framework which we have described so far, we compared our work to current research in representing pathways [63,64]. A pathway is a detailed graphical representation of a biological system, which comprises a set of mutually related events [65]. It integrates pieces of information on biological events scattered in many scientific publications into a coherent system, and thereby facilitates discussion among a large group of biologists and build consensus on what actually happens in a biological system.

The event annotation is intended to be used for development of an ER (event recognition) program. While the results of ER can be used for various NLP-based TM such as intelligent text retrieval, question answering, etc., one of the major challenges is to use them to associate text fragments with relevant part of pathways or to use them to construct semi-automatically pathways. Since events extracted from individual papers have to be integrated into organized networks of events, we need to transform the results of ER to the forms required by pathway models [66].

Research of formalizing pathway representation has made a significant progress in last few years and has reached a consensus on how information on biological events should be represented [63,64], showing how biological events should be represented in a way consistent with the scientific view of a biological system. The consensus actually contrasts with our own event representation. These contrasts highlight the difference between building a biological model, as pathways do, and building a loose biological description, as we find in natural language. From this point of view, the two most significant properties of pathway representations can be summarized as follows:

**(1) Entity-Centered Representation **Pathway representation has become entity-centered, while language organizes information in a predicate-centered manner. That is, pathways are usually organized around state-changes of continuants. The major players in this type of representation, e.g. nodes in a graphical representation, are biological entities which correspond to continuants in specific biological contexts. Events organized around predicates are relegated to mere labels which are attached to links between nodes.

**(2) General Causality **As a typical pathway shows, biological events are intertwined with each other. This makes it difficult, if not impossible, to determine causation, e.g. which event causes which. As a result, pathway representations either eradicate "general" causality from their representations or restrict the relation to a set of limited relations whose underlying mechanisms are well circumscribed.

We discuss each of these issues in detail in the following sections.

### Entity-centered representation

**Figure 5:**
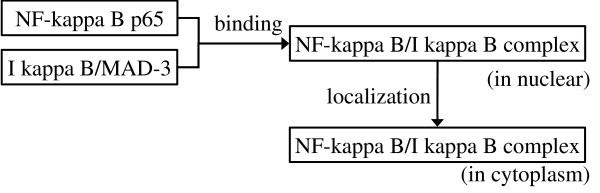
**SBML-style event description for the example in Figure 2**. The nodes denote biological entities. The links denote transitions between different states of entities and correspond to events causing the state transitions.

*Systems Biology Mark-up Language *(SBML) is a framework which is becoming a de facto standard for pathway representation, and which clearly commits to the entity-centered representation [63]. Figure 5 shows the SBML representation for the same set of events as in the previous example, in Figure 2. In this representation, the same continuant, *NF kappa B p65*, appears as three distinct nodes in different biological contexts: one before binding, another after binding, and the third after localization. These three nodes denote instances of the same continuant in different biological contexts. Since these three instances have different properties, it is natural that a pathway representation captures them as different nodes. In this paper we apply definitions introduced in [62], which distinguishes between *continuants *and *instances*. A continuant is an entity which endures, or continues to exist, through time while undergoing different sorts of changes, including changes of location. We use the term *biological entity *to refer to an *instance *of a continuant at a specific time, which is also bound to a specific biological context. The SBML representation is entity-centered since it gives independent status to each of biological entities or instances of the same continuant.

On the other hand, natural language text does not usually make explicit such distinctions among instances of the same continuant with different properties or in different contexts. Consider the example sentence (shown here again for quick reference):

*The binding of I kappa B/MAD 3 to NF-kappa B p65 is sufficient to retarget NF-kappa B p65 from the nucleus to the cytoplasm*.

The two events (the binding and localization events that occur in a sequence) and their relationship are described. Since the sentence is organized around the main predicate "is sufficient to" without any explicit time points, there is no natural way to introduce a new entity (*NF-Kappa B/I kappa B complex *before localization) created by the binding event. The first occurrence of *NF-kappa B p65 *is involved in the binding event, but the expression does not make explicit whether it denotes the entity before or after the event. The same is true for the localization (retargeting) event; since the sentence is organized around the predicate "retarget", the distinction of the entities before and after the retargeting event is not made explicit.

Although such implicitness may be taken as a limitation of natural language as a language for science, it contributes to the easiness and efficiency of communication by language. Human perception of continuants is strong. Even though a continuant may change its properties over the course of an event, it is perceived as the same continuant and expressed as such in language. Such a conception of perpetual existence of continuants strongly influences expressions in language. It may even affect our modes of intuitive understanding and inference. Since continuants recognized as such permeate text, to replace them with distinct entities in different contexts requires a significant reorganization of information in text, and thus makes text annotation extremely difficult.

While the introduction of new entities such as *NF-kappa B/I kappa B complex in nuclear *or *NF-kappa B/I kappa B complex in cytoplasm *may improve the explicitness of pathway representations, in event annotation it is likely to introduce different interpretations by individual biologists. Interpretations which are not properly bound to expressions in text are one of the major causes of inter-annotator discrepancy. As we saw in Section **An example of event annotation**, we have two textual spans with the same expression *NF-kappa B/p65*, but with different Ids. The existence of these two distinguished entities is supported by evidence in text, and will facilitate the transformation from a textual description of the event to a more biology-oriented representation. However, no distinctions which lack explicit textual evidence should be made in the annotation.

### General causality

Representation of General causality is highly related with the treatment of another controversial concept, "Agency." Agency, like causality, is basically an epistemological concept which presupposes that a participant with intention is involved in the event. Among the two major roles, Agent (deep subject) and Theme (deep object), which linguists normally use in the semantic representation of an event, involvement of the Agent in an event is much more tenuous than that of the Theme. In particular, verbs such as "raise," "activate", and "inhibit" which, by themselves, do not specify what actions are taken by their agents, pose special difficulties in semantic analysis.

The sentence "Mary hurt John," for example, can be interpreted as "Mary did something" which resulted in "John being hurt [67]." The sentence explicitly states the *getting hurt *event, and the involvement of John (Theme) is obvious since John is affected by the event. On the other hand, the actual event in which Mary (Agent) is involved is unstated, and the connection between Mary (Agent) and the *getting hurt *event is only causality: whatever Mary did, it caused John to get hurt. In this analysis, verbs like "hurt" are taken to express a causal relationship between unspecified actions, taken by the Agent, and the event which explicitly involves the Theme.

This analysis provides us with a principled way of treating verbs such as "activate," "promote," "inhibit," and "induce." In the domain we are dealing with, there are no Agents with intention except for *Artificial_Process*. We therefore treat these verbs simply as expressions of causality. Consider the following three sentences:

• *Expression of LMP1 in host cells ***activates ***NF-kappa B*.

• *LMP1 needs only 11 amino acids to ***activate ***NF-kappa B*.

• *All six B-cell lines tested showed NF-kappa B activation ***in response to ***LMP1 expression*.

**Figure 6:**
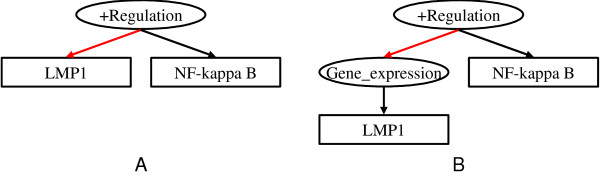
**Graph representations of events about "LMP1 to activate NF-kappa B"**. (A) expresses the event "LMP1 activates NF-kappa B", and (B) expresses the event "expression of LMP1 activates NF-kappa B". Biological implication of the two expressions is equivalent, i.e. since LMP1 activates NF-kappa B, physical manifestation of LMP1, of course, activates NF-kappa B.

These three sentences show the variety of ways in which an event and its causes can be linked in text. The last sentence expresses the causal relationship between the two events (*Activation of NF-kappa B *and *Expression of LMP1*) by linking them with "in response to", while the other two sentences use the verb "activate" to express the causal relation. In addition, the first sentence expresses the cause as an event ("LMP1 expression"), while the second sentence expresses it as an entity ("LMP1"). These two expressions differ on the surface, but they are related in meaning. In our representation, activation of a protein is classified as a Positive_regulation event, following the definition in GO. Such regulation events can have causes, which are other events. Hence, in the first sentence, the event *Expression of LMP1 *can be represented as a cause of the event *Activation *(See Figure 6B). In the second sentence, the protein "LMP1" is directly linked as a cause of *Activation *(See Figure 6A). Equivalence between the two expressions can be recognized by applying a rule of entailment: "If a protein positively regulates an event, physical manifestation of the protein will cause the event."

In contrast to these textual expressions of causality, biology-oriented representations like SBML pathways do not represent causality among events explicitly. Instead, a sequence of state changes of biological entities is represented in a network. A set of biological entities in the upstream of a network is linked with other biological entities in the downstream, whose states change. Causality is represented implicitly by linked paths between entities in upstreams and downstreams. In such a representation, LPM1 would be located on the upstream, with active NF kappa B in the downstream.

**Figure 7:**
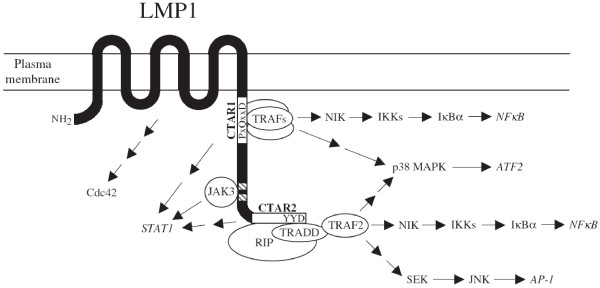
**Molecular interactions and signaling pathways engaged by LMP1**. LMP1 is involved in the activation of NFkB. Even though it has to get through a complex path for the role of LMP1 to take effect on the activation of NFkB, in natural language text, the involvement of LMP1 for the activation of NFkB is often simply written as "LMP1 activates NFkB." Reprinted from [68], Copyright 2001, with permission from Elsevier.

However the pathway representation makes other relationships even more explicit than they usually are in text. For example, the second sentence given above suggests that LPM1 has a binding site of 11 amino acids for an unspecified adaptor protein. No concrete adaptor proteins were mentioned in the abstract where this sentence appears. However, a review paper [68] constructed a partial pathway (Figure 7) in which the adaptor protein was identified as TRADD. This information came from other publications, and the author of the review paper integrated such pieces of information scattered in the literature, in order to create a pathway. Furthermore, the resulting pathway indicates that a long sequence of biological entities and their state changes intervene between LPM1 and activated NF kappa B. The linked path involves the adaptor protein TRADD, NIK, IKK, and others, and finally reaches activated NF kappa B. This is in contrast to the three sentences shown above, which gloss over the linked path by simply expressing that "expression of LPM1" causes "activation of NF-kappa B."

As these examples show, causality expressions are convenient since they allow authors to describe relations among events without explaining the details of underlying mechanisms. Authors may want to leave such explanations out of a publication when they are not relevant or, in some cases, since the authors may not know these underlying mechanisms. For all of these reasons, expressions for causation are pervasive in text. Several more examples are given below:

• *Expression of LMP1 ***activates ***transcription from p50/p65- and c-Rel-responsive promoters*.

• *Expression of LMP1 in EBV-negative nasopharyngeal epithelial cells ***induced ***COX-2 expression*.

• *Inhibition of NF-kappa B in T-lineage cells ***leads to ***a dramatic decrease in cell proliferation*.

• *Overexpression of TRAMP ***leads to ***two major responses, NF-kappaB activation and apoptosis*.

• *Apoptosis can occur ***after ***Bcl-2 phosphorylation*.

In response to the omnipresence of causal expressions in natural language, we have chosen to make causality explicit in our event representation. In addition to expressions like "is sufficient to" and "in response to", verbs such as "induce," "promote," "activate," and "lead to" are treated as expressions of causal relationships between events. Note also that temporal expressions such as "after" are interpreted in some contexts as Causal in our representation.

### Biological annotation and quality control

Before the actual event annotation, we performed a preliminary annotation with a loosely defined annotation scheme. We first gave annotators a set of GO classes with their definitions, and asked each of them to annotate the same set of abstracts. As mentioned previously, we did not restrict these annotations to staying within the boundaries of linguistic structures such as constituent or predicate-argument structure. For example, biologists identified events in expressions such as *the inhibitory effect of CaM-K II on IL-2 promoter *(See Section **Linguistic clues and event classes**). They often saw causal relations among events in temporal sequences such as *apoptosis can occur after Bcl-2 phosphorylation*. They tend to ignore or abstract away from certain linguistic structures. They simply decompose "A activates B as well as C" into two events, "A activates B" and "A activates C". Some adjectives are treated as causes, as in *mitogenic activation *and *thermal activation*, while certain adverbs such as *transcriptionally *in *A upregulates transcriptionally B *are taken to signal events. Our annotators identified two events, upregulation and transcription, in this sentence.

Interesting though they were, the preliminary results of annotation also showed the difficulty of the biological annotation. That is, since it relied on interpretation by individual biologists without specific annotation guides, inter-annotator discrepancies were much larger than we had expected. As a result we developed several techniques for a more sophisticated annotation methodology, which improved inter-annotator agreement.

First, biological annotation inevitably involves interpretation based on background knowledge and information from context. However, these are the two main factors which lead to discrepancies. We had to introduce a principle of annotation to curb the effects of these factors (**Text-bound Annotation**). Second, we had to give very clear guidance on the scope of annotation. This principle guides what types of information should be annotated and what types should not (**Single-facet Annotation**). Finally, we needed careful verification of annotation results. In particular, we found Cross Validation between event and term classes very effective for finding anomalies and cleaning up annotations (**Semantic Typing and Cross Validation**).

The environment for annotation work also played a crucial role in quality control. To share experiences, in particular, reviewing previously annotated text from different annotators became essential for maintaining homogeneity of annotation. The coordinator of annotation organized weekly meetings with the annotators and involved them closely in the adjudication process. We also developed a tool (XConc) for multi-layered annotation. The environment of annotation will be described in the **Methods **Section. Text-bound Annotation, Single-facet Annotation, and Semantic Typing are discussed in the following sections.

#### Text-bound Annotation

The first key principle which we established for reducing annotator discrepancies is called Text-bound Annotation. It can be described simply as follows:

*Associate all annotations with actual expressions in text*.

A similar principle was used in the annotation of Bioinfer [56]. As in BioInfer, we do not allow annotators to annotate an event unless an expression mentioning the event type appears in the text. However in our attempt we deliberately dissociate annotation from linguistic structures, and events in our annotation are not necessarily organized around verbs. That is, an event does not necessarily correspond to a constituent such as a clause or phrase, governed by a verb. Expressions which indicate occurrences of an event and expressions which describe its participants (arguments) can be scattered throughout a sentence without constituting a single constituent in the linguistic structure. Nonetheless, such expressions must be provided for each annotation, and we refer to them as "clue words" or "clue expressions". This principle ensures that each annotation is grounded in textual evidence, and that annotations are not the result of unbounded interpretation by individual annotators. It applies even when the annotator could infer the existence of an event from context (See Section **Linguistic clues and event classes**).

We also aligned our annotations to single sentences. That is, all evidence attached to an event should come from the same sentence. There are some cases in which slots for arguments are filled by anaphoric expressions such as pronouns, definite noun phrases and noun phrases with demonstrative determiners (such as *this *or *these*). Only in such cases were annotators allowed to expand the scope of annotation, identifying textual expressions outside the current sentence to fill the argument slots. Even in these cases, expansion of scope is explicitly indicated by a special link (Co-Ref link), which associates the anaphoric expression inside the sentence with the entity outside.

The goal of these restrictions is to prohibit annotators from introducing entities or events which lack textual clues in the same sentences. This does not imply that annotation was performed sentence by sentence, without considering context. On the contrary, the annotators were encouraged to use the document context for disambiguation. Consider the following examples:

• *In addition, forced expression of GATA3 potentiated the induction of RALDH2 by TAL1 and LMO, and these three factors ***formed a complex ***in vivo* (Figure 4D).

• *Furthermore, a TAL1 mutant not binding to DNA also activated the transcription of RALDH2 in the presence of LMO and GATA3*.

• *In contrast, in vivo footprints on GT (CACCC) motifs differed between the cells expressing the fetal or the adult globin program*.

In the first of these sentences, an annotator has to disambiguate the anaphoric expression *these three factors*. Without context, it can refer to either of the two sets of entities, (*TAL1*, *LMO*, *RALDH2*) or (*TAL1*, *LMO*, *GATA3*). However the second sentence provides enough context for the annotator to identify the third element in the set as *GATA3*, not *RALDH2*. It is important to note that this type of interpretation still adheres to our principle of Text-bound Annotation, because it relies on textual evidence in the same sentence: namely, the anaphoric expression.

On the other hand, although *footprints *in the third sentence indicates a DNA-binding event, implying the presence of a protein which is bound, there are no textual clues in the sentence to indicate the existence of such a protein. In such cases, annotators were not permitted to represent this protein (the hypothesized Theme of binding) in annotation, even if they could identify the missing element from context. As a result, we see quite a few events in our annotation which lack necessary arguments (See **Results and discussion **Section). To fill them from context remains a topic for future work, since this would require carefully calibrated guidelines to ensure inter-annotator agreement.

#### Single-facet Annotation

Our second key principle for reducing annotator discrepancies is called Single-facet Annotation. It is described as follows:

*Keep the view point for annotation as simple and focused as possible*.

Consider the following sentence:

*Calcineurin acts in synergy with PMA to inactivate I kappa B/MAD3, an inhibitor of NF-kappa B*.

One annotator identified a single event in this sentence, which was *Inactivation of I kappa B/MAD3 by Cacineurin*. However, another annotator claimed that the sentence conveys additional biologically important information: that calcineurin actually enables NF-kappa B to be activated by inactivating I kappa B/MAD, which inhibits NF-kappa B. For her, the expression "I kappa B/MAD3, an inhibitor of NF-kappa B" indicated another event: *Inhibition of NF-kappa B by I kappa B/MAD3*. This is a typical discrepancy caused by the multi-faceted nature of information in text.

When we see the sentence from the view point of events and their relationships, we interpret the sentence in the same way as the second annotator. That is, we consider every expression in the sentence as possible evidence of an event, even in cases where there is no explicit verb, as in "I kappa B/MAD3, an inhibitor of NF-kappa B".

On the other hand, the first annotator read the sentence from a rather general, non-focused view. She used a generic interpretation of the linguistic device of apposition, so she interpreted the same expression as a static IS-A relation, i.e. *I kappa B/MAD3 *IS-A *inhibitor of NF-kappa B*.

The goal of Single-facet Annotation is to reduce such discrepancies by defining one aspect of text as the focus of annotation. In our annotation, we asked annotators to examine text from the focused view point of events and their relationships. We gave each annotator a list of event classes from GO (the 35 event classes we chose) and asked them to identify as many events and their relations as possible in every sentence, within the limit imposed by Text-bound Annotation. We call this **Event-centered Annotation **as an instance of Single-facet Annotation.

Event-centered Annotation not only reduced annotator discrepancy but also contributed to the identification of a diverse vocabulary of event-related expressions. This is a secondary feature of Single-facet Annotation. As the annotation example above shows, focusing our interpretation on one facet of text, like the expression of events and their relationships, allows us to ignore the constraints that are usually imposed by other facets, like linguistic constituent structure. When we instruct annotators to examine every part of a sentence with respect to its role in an event, they are able to ascribe event-related meanings to parts of the sentence that cross constituent boundaries and that do not conform to predictable predicate-argument structures. Table 1 shows examples which were identified as *Inhibition *events by the annotators. These examples demonstrate the wide variety of expressions that can be interpreted as events under the principle of Single-facet, Event-centered Annotation.

**Table 1:** Linguistic realization of the word "inhibit" in various context

**Sentences/Clauses**
• *Our results indicate that ESI **inhibits **NF-kappaB activation*.
• *We investigated the capacity of EC to **inhibit **NF-kappaB activation*.
• *Baicalin was shown to **inhibit **the NF-kappaB cascade*
• *Montelukast prevents the decrease of interleukin-10 and **inhibits **NF-kappaB activation*.

**Nominalization**

• ***Inhibition **of ERK1/2 abrogated genistein-mediated NfkappaB activation*
• *Combined pharmacologic PARP **inhibition **and irradiation with 15 Gy significantly reduced neointimal hyperplasia*

**Derived nouns and Pre-nominal modifiers**

• *Calcineurin acts in synergy with PMA to inactivate I kappa B/MAD3, an ****inhibitor ****of NF-kappa B*.
• *In this report we have examined the efficacy of the gold compound AuTG as an **inhibitor **of HIV replication*.
• *The **inhibitory **effect of CaM-K II on IL-2 promoter was associated with decreased transcription of its AP-1 and NF-AT transactivating pathways*.
• *Western blotting analysis indicated that E3330 inhibited degradation of I kappa B-alpha, which is an **inhibitory **protein of NF-kappa B*.

**Complex expressions, Constructions with weak verbs**

• *These findings establish that Rel can function as an **inhibitor **of gene expression and is required by T lymphocytes for production of IL-3 and GM-CSF*.

Hence, these principles work together to bound the interpretations given by annotators. Single-facet Annotation, in particular Event-centered Annotation, forces annotators to identify events, rather than static semantic relationships (IS-A, for example), or syntactic features. According to this principle, they should annotate as many events as possible. The principle of Text-bound Annotation gives this process a well-defined stopping criterion: "As many as possible" means precisely the number of events that can be linked with textual evidence, or clue words, from the same sentence.

#### Semantic Typing and Cross Validation

The GENIA event classes correspond to biologically homogeneous classes. This property is manifested in the homogeneity across entities (GENIA terms) which appear as arguments for the events in a given class. Although the relationship between GENIA term and event classes is not so straightforward (See Section **Distribution of semantic types**), semantic homogeneity of these arguments has been useful for Cross Validation of term and event annotations.

When only a relatively small number of instances of event annotations contain entities from specific term classes, either the term annotation or the event annotation may be wrong.

For example, after an initial phase of annotation, for the event class Gene_expression, we found the following patterns suspicious, since their rates of occurrence are very small:

• Gene_expression of Peptide (5 instances)

• Gene_expression of Nucleotide (2 instances)

• Gene_expression of Lipid (1 instances)

After verification, 4 annotation instances of the first case (Peptide) were accepted as correct annotations. The others were errors in which the wrong terms Ids had been given for the arguments. We added a new functionality to the annotation tool, XConc, to prevent the same errors from occurring again.

We also found many *Binding *events where two instances of DNA were annotated as Themes. However, the annotation coordinator was suspicious, thinking that DNA-DNA binding should be rare in our domain (transcription factors in human blood cells). When those instances were checked at an adjudication meeting, it turned out that there had been quite a few errors in term annotation. At the same time, they found that a few instances of DNA Metabolism had been wrongly annotated as *Binding*. An example is given below:

• *In the T cell line CTLL2, ligation of kit/IL-4R alpha induces cellular proliferation*.

Ligation can be considered a type of binding. However, in GO, it is classified under DNA Metabolism. One annotator was not aware of this. Through our process of Semantic Typing and Cross Validation, we were able to find and correct the resulting inconsistencies. In Table 2 (of which a detailed description is given in Section **Distribution of semantic types**), 31 instances of DNA-DNA Binding still remain, but all of them are instances of Binding by a DNA-probe, which can appear in the domain of the GENIA corpus.

**Table 2:** Distribution of theme classes for Transcription, Translation, Gene_expression and Binding events

**Transcription**	**Translation**	**Gene_expression**	**Binding**
DNA (538)	Protein (34)	Protein (2,569)	Protein DNA (1,186)
RNA (334)	DNA (7)	DNA (904)	Protein Protein (611)
Protein (291)	Virus (1)	Virus (47)	Protein (288)
Virus (38)		RNA (30)	DNA (77)
*No theme (16)		Peptide (4)	Other_organic_compound Protein (58)
			Protein Lipid (48)
			DNA DNA (31)
			Polynucleotide Protein (22)
			Protein Protein Protein (10)
			DNA Protein Protein (8)
			...

The lists for the event classes, *Transcription*, *Translation *and *Gene_expression *are complete. For the event class Binding, the 10 most frequent theme patterns are shown. Note that *Binding *events are allowed to be annotated with more than one themes.

### Annotation results

As a result of completing this stage of event annotation, we were able to examine some important distributions in detail. First, the distribution of Linguistic clue words with regard to event classes. Second, the distribution of Semantic Event classes themselves. We describe each of these in the following sections.

#### Linguistic clues and event classes

Clue words are important in our framework not only because they help enforce the principle of Text-bound Annotation but also because they can be used in the next stage of our work, i.e. development of ER (Event Recognition) programs. They can be used as features for Machine Learners or as key words in rules for ER. However, the distribution of clue expressions indicates the kinds of difficulties which an ER program will have to resolve. In a similar way as NER (Named Entity Recognition), ER has to deal with difficulties caused by the ambiguity and diversity of language.

Table 3 shows three representative event classes with the distribution of their linguistic clues. The distribution suggests that diverse words with different POS and syntactic structures are used to describe the same events. While some clue expressions such as "transcription" or "transcribe," "translocation," "secretion," and "cross-linking" unambiguously denote single event classes, other clues such as "engage," "recognize," and "associate" are general and ambiguous.

**Table 3:** Clue expressions for some event classes

**Regulation**	**Binding**	**Localization**
regulation [of] (178)	binding (256)	translocation [of] (88)
involved [in] (139)	binding [to] (123)	translocation (58)
effects [of] [on] (137)	binding [of] [to] (120)	secretion (57)
role [of] [in] (124)	binding [of] (114)	release (49)
dependent (106)	bind [to] (106)	secretion [of] (33)
regulated [by] (102)	bind (84)	secreted (23)
regulate (101)	binds [to] (83)	release [of] (23)
effect [of] [on] (98)	bound [to] (68)	mobilization (16)
affect (94)	binding activity (67)	localization [of] (16)
regulating (75)	interact [with] (57)	present (13)
effect [on] (72)	binds (48)	uptake (12)
regulated (66)	bound (44)	import [of] (12)
regulation [of] [by] (64)	interacts [with] (42)	released (11)
regulates (61)	associated [with] (37)	localization (10)
control (50)	interaction [of] [with] (34)	appearance [of] (9)
affected [by] (50)	cross-linking (34)	secreting (8)
controlled [by] (47)	interaction [with] (29)	mobilization [of] (8)
control [of] (45)	interaction (22)	localized (8)
regulation (40)	binding activity [of] (22)	uptake [of] (7)
plays * role [in] (35)	ligation (21)	translocated (7)
affected (34)	binding [for] (20)	translocate (5)
transcriptional regulation [of] (33)	interactions (19)	mobilized (5)
response [to] (33)	recognized [by] (17)	import (5)
effects [on] (33)	engagement (16)	distributed (5)
dependent [on] (32)	cross-linking [of] (15)	co-localization [with] (5)
play * role [in] (31)	association [with] (15)	translocates [as a result of] (4)
involvement [of] [in] (31)	bound [by] (14)	translocates (4)
modulating (30)	recognizes (12)	secrete (4)
responsible [for] (28)	interacted [with] (11)	migrating (4)
effect (28)	interactions [with] (10)	accumulation [of] (4)
changes [in] (27)	binding [by] (10)	shuttling [of] (3)
controls (25)	association [of] [with] (10)	sequestered [via] (3)
role [for] [in] (24)	associates [with] (10)	presence (3)
are key regulators [of] (24)	complexed [with] (9)	imported (3)
modulate (23)	ligation [of] (8)	expression [of] (3)
independent (22)	engagement [of] (8)	delivery [of] (3)
role [of] (21)	binding activities [of] (8)	topography (2)
regulators [of] (21)	associate [with] (8)	stored (2)
controlling (21)	linked [to] (7)	sequestered [by] (2)
affecting (21)	interaction [between] (7)	sequester (2)
...	...	...

The 40 most frequently observed clue expressions for each of three event classes, *Regulation*, *Binding *and *Localization*. The asterisk sign (*) indicates discontinuity at that position. Words in square brackets are functional words which appear together with clue expressions to form linguistic patterns to connect the clue expressions to their themes and causes.

The following two sentences show how the verb "associate" can refer to two different event classes.

• *In coimmunoprecipitation experiments using transfected COS cells, GATA-1 and ER ***associate ***in a ligand-dependent manner*. [Binding]

• *The induction of these genes is ***associated ***with interleukin-2 (IL-2)-induced T-cell proliferation*. [Correlation]

In addition, while the event class of Binding has many specific clue expressions such as "bind", "interact", and "ligation", general expressions which are used for other event classes also appear. Examples are given below:

• *CTLA-4 ***engagement ***by mAbs inhibits IL-2 production and proliferation upon T cell activation*.

• *The GM-kappa B sequence is ***recognized ***by NF-kappa B, which is mainly induced by PMA*.

These ambiguous verbs with broad meanings would cause difficulties for event extraction programs. Even seemingly non-problematic verbs, such as "activate" or "bind," are ambiguous from the biological view point. In the current event-annotated corpus, there are 1,785 occurrences of "activate" which are annotated as *Positive_regulation*, while 496 occurrences are annotated as *Physiological_process*. However, uses of the word "activate" labeled with *Physiological_process *convey the same meaning as uses which are labeled *Positive_regulation*, i.e. either the number of the entity in the Theme increases or the function of the Theme is materialized. The ambiguity is purely due to the organization of the class hierarchy of GO. Events denoted by "bind" require a similar distinction. The term is sometimes used to refer to *Cell_adhesion*, which is a separate class from *Binding *in GO. However a larger proportion of occurrences of "bind" are still annotated as *Binding *events.

• *Induction of cytokine expression in leukocytes by ***binding ***of thrombin-stimulated platelets*. [Binding]

• *Combinations of hypoxia and LPS significantly increased lymphocyte ***binding**. [Cell_adhesion]

These ambiguities are not ambiguities of the meaning of the words themselves. They share the same linguistic core meanings. Instead, their ambiguities come from the biological heterogeneity of the events that these expressions denote. In these cases, annotators have to check the semantic classes of Theme in the term ontology for the correct classification of these events. The annotation guidelines list such confusing cases explicitly.

The class of regulatory events has the most diverse clue expressions. This is partly because, unlike other event classes, this class denotes relationships among events or processes. As noted before, the class *Regulation *which we use for event annotation covers a much wider range of relations than its counterpart in GO. We use it to denote general causal relationships among events. This may also contribute the diversity of clue expressions. In GO, regulatory events are sub-classified further. One may argue that subclassification of regulatory events leads to more uniform clue expressions for subclasses. This remains to be examined, but since most of the clue expressions for this class are general terms such as "regulate," "dependent," or "affect," we doubt that this is the case.

#### Distribution of semantic types

Table 2 shows the distribution of term classes which appear as Themes of four events: *Transcription*, *Translation*, *Gene_expression *and *Binding*. Reflecting the nature of the event classes, the first three events, Transcription, Translation, and Gene expression, appear with a small, concentrated list of term classes as their Themes. This is in contrast to the long list of term classes that appear as the Theme of Binding. The first three classes are all related to gene expression, which consists of two micro events of Transcription and Translation.

As we expect, gene-related entities like DNA, RNA, and proteins are identified as possible Themes of the first three classes. The same is true of viruses, which often have genes expressed inside human bodies. In addition, we see a small number of occurrences of peptides which are gene products (e.g. insulin, GH). However, closer examination reveals interesting and rather convoluted phenomena. From the biological point of view, *Transcription *is the first step of *Gene_expression*, transcribing DNA to RNA. From a naive predicate-centered view, this means that DNA appears as the Theme of the event, and RNA appears as the Location. Accordingly, as Table 2 shows, the majority (538) of Themes in our annotated *Transcription *events are instances of DNA. The following is an example of such a sentence:

*The Ca(2+)-dependent factor NF-ATP plays a key role in the inducible transcription of both these lymphokine genes*.

On the other hand, a transcription event can also be described from the view point of what is produced as a result. In this case, the Theme is RNA, i.e. what is expressed by *Transcription*. The following is an example:

*These B cells expressed p40 and p35 mRNA, and phorbol myristate acetate (PMA) stimulation strongly enhanced p40 and p70 production*.

The frequency of this type of expression, in which RNA was annotated as the Theme, is also high (334). Since linguistic expressions do not distinguish entities before or after an event, an entity can be described as a Theme in either of its states, before or after the event.

More interestingly, we observed quite a large number (291) of occurrences of Protein as the Theme of *Transcription*. The following is one of the typical contexts in which this occurs:

*YM268 facilitated the insulin-stimulated triglyceride accumulation in 3T3-L1 adipocytes and increased the mRNA expression of fatty acid-binding protein*.

Although "fatty acid-binding protein" has been annotated as a Protein, what is actually transcribed is the genomic information for the protein. In a specific context (i.e. transcription, translation or gene expression), the physical form (or the container) of the genomic information of the protein is obvious. Thus sometimes, the Theme is rather less strictly described in text.

This phenomenon is related with our perception of continuants (like proteins) and with systematic metonymy [69,70], which permeates language. For an example of systematic metonymy, consider the following sentence given in everyday language:

*The picture was developed, printed and sent to him*.

Precisely speaking, what was developed is actually the film containing the picture, what was printed is the content of the picture (an image), and what was sent was the printed picture (physical manifestation of the image). The same expression "picture" is used in different contexts of development, printing, and delivery. Depending on the context, the proper interpretation is taken by the reader.

Similar phenomena are frequently observed in our domain. In the following example, the three terms "JunB", "FosB" and "c-Fos" are used to refer to genes in the context of transcription, and then used to refer to the corresponding proteins in the context of DNA binding.

...* which correlates with an absence of JunB, FosB, and c-Fos transcription, as well as an absence of their DNA-binding activity*.

In the current release of the GENIA event corpus, the term and event annotations will be kept as they are. However, these phenomena will have to be carefully studied to design a new annotation scheme. The scheme should be able to accommodate both the context-dependent nature of term semantic classes, and the context-independent nature of the classes of continuants.

Some *Transcription *events are annotated without any Theme. This is because transcription is often mentioned as a function of a protein as follows:

*Transcriptional activity of p105 is also increased in infected cells and is also mediated by NF-kappa B through a specific kappa B motif*.

Because our Single-facet Annotation principle focuses on events and their relations, the function of a protein is interpreted as a potential event regulated by the protein. Hence, the expression "Transcriptional activity of p105" in the above sentence is paraphrased as "transcription event regulated by p105". However, since the original sentence is different (e.g. the function of the protein), the Theme of the event (what is transcribed) is out of scope and not mentioned. The same phenomena are observed in *Regulation*, *Positive_regulation *and *Negative_regulation *events in Table 4.

**Table 4:** Distribution of theme classes for Regulation events

**Regulation**	**Positive_regulation**	**Negative_regulation**
Positive_regulation (702)	Protein (2,413)	Positive_regulation (1,505)
Gene_expression (586)	Gene_expression (1,560)	Protein (595)
Protein (453)	Positive_regulation (1,499)	Gene_expression (465)
DNA (426)	DNA (902)	Binding (269)
Transcription (239)	Transcription (632)	DNA (187)
Regulation (237)	Binding (446)	Transcription (164)
*No theme (192)	Negative_regulation (356)	Regulation (126)
Binding (133)	Cellular_phy_process (345)	Localization (126)
Physiological_process (120)	Localization (341)	Cellular_phy_process (122)
Negative_regulation (108)	Regulation (309)	Negative_regulation (121)
Cell_differentiation (106)	*No theme (277)	Viral_life_cycle (94)
Cellular_phy_process (95)	RNA (268)	*No theme (76)
Viral_life_cycle (65)	Cell_differentiation (220)	Physiological_process (57)
Cell (61)	Protein_phosphorylation (214)	Cell_differentiation (50)
Localization (60)	Physiological_process (154)	Cell (50)
Cell_communication (36)	Viral_life_cycle (141)	RNA (48)
RNA (31)	Cell_adhesion (86)	Cell_adhesion (43)
Protein_phosphorylation (26)	Protein_catabolism (84)	Cell_communication (40)
Cell_adhesion (18)	Biological_process (74)	Protein_catabolism (34)
Virus (17)	Cell_communication (71)	Protein_phosphorylation (29)
...	...	...

The 20 most frequent theme classes are shown for each of the *Regulation*, *Positive_regulation *and *Negative_regulation *event type. Note that events of *Regulation *type are allowed to be annotated with another event as their theme.

The fact that a large number of events without any Theme (16 in Transcription, 192 in Regulation, 277 in Positive_regulation, 76 in Negative_regulation) were annotated indicates that our Single-facet Annotation worked as we hoped. That is, taking an Event-centered view of each sentence caused the annotators to identify every event mentioned in the text, including the main event indicated explicitly by the author as well as events which are described peripherally, with little additional detail.

Missing Themes in Binding events described in the **Semantic Typing **Section are same in nature. The annotators identified DNA binding in sentences such as

*A ***footprint ***was visible over this region of he c-myb5' flanking sequence in activated T-cell but not in unactivated T-cell*

One can safely assume the existence of another Theme of binding, which is the protein that left the footprint, but there was no mention of this protein in the text.

Table 4 and 5 show the type distribution of Themes and Causes of regulatory events, respectively, while Table 6 shows a breakdown of the *Positive*_ and *Negative_regulation *which appear as Causes of regulatory events. The type distribution of Themes systematically corresponds to the subclassification of *Regulation *in GO. This means that, if we recognized basic event types, we could further subclassify them by rather simple rules referring to the types of their arguments. The only exceptions are the cases in which terms, instead of events, occupy the Theme. In these cases, ambiguity remains as to whether Positive regulation means increasing their amounts or enabling their functions.

**Table 5:** Distribution of cause classes for Regulation events

**Causes of Regulation**
Protein (5,797)
*No cause (4,184)
Other_organic_compound (2,398)
Positive_regulation (1,291) (See Table 6 for breakdown.)
DNA (1,045)
Lipid (713)
Negative_regulation (630) (See Table 6 for breakdown.)
Physiological_process (601)
Binding (577)
Artificial_process (448)
Mutagenesis (348)
Gene expression (322)
...

The 12 most frequent cause classes for Regulation (without differentiating *Positive*_ or *Negative_regulation *events).

**Table 6:** Breakdown of causes in Positive_ and Negative_regulation

**Positive regulation (1,291)**	**Negative regulation (630)**
Protein (405)	Protein (200)
Gene_expression (218)	Positive_regulation (69)
DNA (46)	Gene_expression (27)
Protein_amino_acid_phosphorylation (25)	DNA (24)
Localization (23)	Localization (19)
RNA (18)	Protein_amino_acid_phosphorylation
...	...

The *Positive_regulation *and *Negative_regulation *events which appear as causes in Table 5 are further classified in a more detail according to their themes.

The type distribution of Causes also shows some interesting tendencies. A large portion of the Causes are proteins (5,797). While these are topics beyond the scope of this paper, we are now formulating entailment rules by which we can transform all complex cases, such *Positive_regulation *of *Protein *(405) and *Gene_expression *of *Protein *(218), into *Protein*, or vice versa. We expect that a set of such entailment rules will make our representation framework capable of handling variable granularity and underspecification of information, which are essential properties of natural language.

## Conclusion

In the bio-medical domain, event or relation annotation has not been conducted on a large scale, though it is recognized an important step towards advanced NLP-based TM. There are several known difficulties for successful completion of event annotation. We have to first define an annotation scheme, and then perform a large amount of annotations consistently.

In this paper, we first discussed some of basic characteristics of information encoded in text such as underspecification, variable granularity of information, and predicate-centered description, which are reflected in our design of annotation scheme. Then we presented our strategies for maintaining the quality of annotation, including the principles of Text-bound Annotation and Single-facet Annotation, as well as Cross Validation by Semantic Typing.

There remain discrepancies in annotation, in particular, in the annotations of semantic roles other than Cause and Theme. We also need to establish a more theoretically sound framework to treat the relationship between term and event classes in annotation.

In spite of these remaining problems, as the first phase, the current version of event annotation is complete in its own right. The quality and the size of the annotated corpus make it one of the best and largest, compared to similar attempts. In combination with the existing annotations on the GENIA corpus, the annotation discussed in this paper will contribute to further progress in NLP-based TM activities, such as event extraction, intelligent information retrieval, semantic enrichment of text, and integration of text information with pathway databases. The event-annotated corpus and the annotation guidelines will be made publicly available in XML at the homepage of GENIA Project [71].

## Methods

### Annotation procedure

We started our annotation work in May, 2005 with one coordinator (a biologist working full-time on this project) and three graduate students in molecular biology. The initial phase of three months was for exploratory annotation, in which three annotators were given the same set of abstracts to annotate and a rather simple annotation manual. The manual was provided by the Caderige project [59].

Inspection of the first annotation results revealed greater discrepancies than we had expected. This is partly because the manual provided by the French group was intended for annotation of text on "Bacillus subtilis and transcription", and there were many phenomena that were not covered. More seriously, biologists made overly-subjective interpretations using their own background knowledge and by referring to context.

Based on these results, we revised the manual substantially and started the annotation work in earnest in December, 2005. We also found that sharing experience and discussing specific annotation examples among the annotators is crucial for maintaining quality. Therefore, we decided to organize frequent meetings between the coordinator and the annotators. The meetings continued regularly, once every week, until the end of the first phase. The coordinator and the annotators were jointly involved in the adjudication process of problematic cases. Special databases and annotation software (XConc) were developed to maintain the results of adjudication and facilitate flexible retrieval of annotation results, for reference.

Since we had already finished the term annotation, we were able to check semantic homogeneity of arguments in the annotated events. We found that examination of the distribution of term classes used as arguments was effective for finding anomalies in annotation, and we made it a regular practice at the meetings to discover problematic cases.

There have been changes of annotators. Whenever a new annotator joined, we trained her/him by using previously annotated examples, with a constantly-revised manual. On average, five part-time annotators (graduate students), one senior coordinator and one junior coordinator have been involved in annotation throughout the whole period of 1.5 years.

### Annotated information

While the current annotation focuses on identification of event classes, along with clue expressions for classes and fillers of the two major roles Cause and Theme, we annotated other semantic roles as well. These role annotations correspond to semantic role assignments for the complements and adjuncts of verbs, as seen in linguistics-based annotation efforts such as PropBank. Except for some event classes that internally involve locations, these semantic roles capture the biological context where an event takes place. Examples of these additional semantic role annotations are shown in Table 7.

**Table 7:** Semantic role types and their annotation instances

**clueLoc**	**clueTime**	**clueExperiment**
nuclear (135)	early (15)	electrophoretic mobility shift assays (13)
in t cells (106)	subsequent (13)	northern blot analysis (9)
in human monocytes (57)	during t-cell activation (8)	electrophoretic mobility shift assay (7)
in monocytes (50)	within 30 min (6)	by electrophoretic mobility shift assays (7)
in b cells (41)	initial (6)	using electrophoretic mobility shift assays (4)
intracellular (38)	during aging (6)	in transient transfection assays (4)
in jurkat cells (34)	during monocytic differentiation (5)	in emsas (4)
in jurkat t cells (33)	simultaneous (4)	in electrophoretic mobility shift assays (4)
in monocytic cells (30)	during the immune response. (4)	site-directed mutagenesis (3)
in t lymphocytes (28)	during erythroid differentiation (4)	nuclear run-on experiments (3)
surface (27)	at 24 hr (4)	in gel mobility shift assays (3)
cells (24)	rapidly (3)	immunoblot analysis (3)
cytoplasmic (21)	for 6 hours (3)	gel-shift analysis (3)
in these cells (20)	for 6 h (3)	emsa (3)
in human t cells (20)	first (3)	cotransfection experiments (3)
t cells (18)	during the immune response (3)	by northern blot analysis (3)
cellular (18)	during the cell cycle (3)	by flow cytometry (3)
in activated t cells (17)	during t cell activation (3)	by electrophoretic mobility shift assay (3)
to the nucleus (16)	during myelopoiesis (3)	western blotting (2)
in the nucleus (16)	during monocyte differentiation (3)	transient transfection experiments (2)
in hela cells (16)	at 8 hr (3)	supershift analysis (2)
in thp-1 cells (15)	24 h (3)	rt-pcr (2)
in b lymphocytes (15)	within 8 hr of infection (2)	northern blot analyses (2)
b cell (15)	within 6 h (2)	northern analysis (2)
in u937 cells (14)	within 4 hours (2)	mutational analysis (2)
in fibroblasts (13)	within 20 min (2)	mutational analyses (2)
b cells (13)	within 2 h (2)	mobility shift assays (2)
transendothelial (12)	second (2)	inhibition studies (2)
t-cell (12)	in a primary t cell response (2)	in transient transfection experiments (2)
into the nucleus (12)	from day 7 to day 14 of culture (2)	in transient assays (2)
...	...	...

Text expressions providing locational (clueLoc), temporal (clueTime) and experimental (clueExperiment) context where biological events take place. The 30 most frequently observed expressions for each type are listed.

### Annotation tools

From time to time, we changed the annotation criteria and the format of annotation. Since such changes had to be reflected in previous annotations, we developed a tool for manual annotation, XConc Suite, which provides annotators with the functions of retrieving and editing existing annotations, as well as functions for creating new annotations.

**Figure 8:**
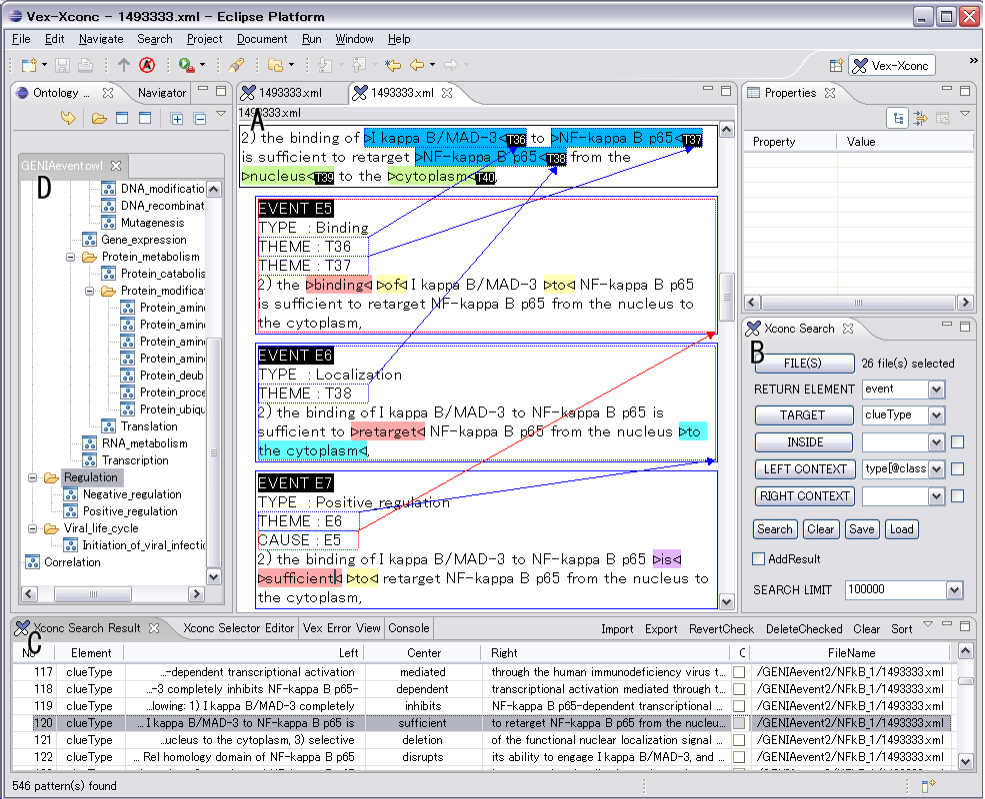
**Screenshot of XConc Suite**. The XConc Suite consists of three plug-ins to Eclipse platform: an XML editor (*A*), a concordancer (*B *is the query editor and *C *is the result view), and an ontology browser (*D*) which support both the editor and the concordancer for the selection of ontology terms.

The XConc (XML-based Concordancer) Suite is an integrated annotation environment providing an XML editor, a concordancer and an ontology browser which all interact with each other. For example, the users can retrieve existing annotations and view the concordance in KWIC (keyword in context) format. Figure 8 shows a screenshot of XConc. The pane in the bottom shows the list of annotation instances of *Regulation *(including its child classes, *Positive_regulation *and *Negative_regulation*). Users can choose an instance from the list in order to open the file containing the annotation, which will automatically locate the cursor on the annotation, so that they can easily make an addition to it.

In the figure, an XML editor in the middle shows an annotation instance from the underlying XML file. The cursor is located in the text span "sufficient" in the bottom of the editor window. This is the result of choosing a specific instance from the annotations list, mentioned above. The XML editor and the concordancer are integrated with the ontology browser (shown in the left of the screenshot). This allows users to select an event or term class (including or excluding its child classes) using the browser, both for annotation creation and annotation search.

The XConc Suite is implemented on top of Eclipse, a widely used, general-purpose software development platform. This provides the XConc Suite with general functionality for software development, including project and file management and version control. A proper version control system, like CVS supported by Eclipse, is particularly crucial for long term software development activities like corpus annotation. Another characteristic feature of the XConc Suite is its flexibility. Since it is developed for general XML applications rather than for a particular format, the annotation schemes and corresponding user interfaces are easily customized by using *DTD *(Document Type Definitions) [72] and *CSS *(Cascade Stylesheets) [73]. XConc has evolved in parallel with actual annotation work. In particular, the following functionalities of XConc were found indispensable for efficient and stable annotation.

1. Functions for Multi-layered Annotation: Our event annotation is based on the term annotation, which was completed previously on the same corpus. Arguments of an event, Cause and Theme, are chosen from already-annotated terms. This reduces discrepancies in terms of selection of text spans for arguments. When appropriate terms were not annotated as such in the term annotation, annotators were required to report to the coordinators.

2. Functions for Ontologies: The two ontologies, the GENIA term ontology and the GENIA event ontology, are represented in OWL, and XConc manages them using Protégé. While we encouraged annotators to use as many leaf concepts as possible in the event annotation, we also gave them guidelines for when they should use broader, less specific concepts. This avoids forced interpretation and thus reduces discrepancies. Tools for navigating through the ontologies were essential for annotators when choosing the appropriate event classes.

3. Functions for Structured Objects: Unlike the term annotation, the event annotation has to deal with the internal structures of an event, such as elements which fill the roles of Theme and Cause. An event can be a role-filler for other events. Flexible functions for assigning Ids to structured objects, and for referring to them by these Ids in subsequent annotations, are indispensable for efficient annotation.

4. Functions for Retrieval: XConc provides functions for retrieval of annotations previously made, based on patterns of annotation tags. To examine previous annotations in similar contexts, especially the ones which have already been adjudicated by the coordinators, is very useful for maintaining homogeneity of annotation.

These functions correspond to ways in which annotators interact with the corpus in the process of performing their work.

## Authors' contributions

JT conceived the original idea and supervised all steps of the work. JDK and TO cooperated in designing the ontology and annotation scheme, with JDK from an Information Science point of view and TO from Biology. JDK developed software tools to support annotation, while TO coordinated the actual annotation by annotators. JDK and JT wrote the manuscript. TO provided examples and revised the manuscript from a biological perspective. All authors read and approved the final manuscript.
